# Enhancement of Mass Transfer Process for Photocatalytic Reduction in Cr(VI) by Electric Field Assistance

**DOI:** 10.3390/ijms25052832

**Published:** 2024-02-29

**Authors:** Xi Feng, Yonghui Lin, Letian Gan, Kaiyuan Zhao, Xiaojun Zhao, Qinhe Pan, Guohua Fu

**Affiliations:** 1 School of Ecology and Environment, Hainan University, Haikou 570228, China; 2 School of Chemistry and Chemical Engineering, Hainan University, Haikou 570228, China; 3 Management School, Hainan University, Haikou 570228, China

**Keywords:** photocatalysis, electric field assisted, PAF-54 Cr(VI) removal, wastewater treatment

## Abstract

The removal of Cr(VI), a highly-toxic heavy metal, from industrial wastewater is a critical issue in water treatment research. Photocatalysis, a promising technology to solve the Cr(VI) pollution problem, requires urgent and continuous improvement to enhance its performance. To address this need, an electric field-assisted photocatalytic system (PCS) was proposed to meet the growing demand for industrial wastewater treatment. Firstly, we selected PAF-54, a nitrogen-rich porous organic polymer, as the PCS’s catalytic material. PAF-54 exhibits a large adsorption capacity (189 mg/g) for Cr(VI) oxyanions through hydrogen bonding and electrostatic interaction. It was then coated on carbon paper (CP) and used as the photocatalytic electrode. The synergy between capacitive deionization (CDI) and photocatalysis significantly promotes the photoreduction of Cr(VI). The photocatalytic performance was enhanced due to the electric field’s influence on the mass transfer process, which could strengthen the enrichment of Cr(VI) oxyanions and the repulsion of Cr(III) cations on the surface of PAF-54/CP electrode. In addition, the PCS system demonstrates excellent recyclability and stability, making it a promising candidate for chromium wastewater treatment.

## 1. Introduction

With the development of the social economy and the increasing demands for a higher quality of life, ecological sustainability has become an inevitable requirement for people [1,2]. However, environmental pollution poses a threat to people’s physical health and life safety, making it a problem that must be addressed in the construction of ecologically livable environments. Chromium(Cr) pollution serves as a typical example as it is a common toxic element that can accumulate in the body and cause a variety of diseases [3,4,5]. Chromium is widely utilized in industrial operations such as mining, electroplating, afterchrome dyeing, steel manufacturing and leather manufacturing [6,7]. Many enterprises in developing countries often accumulate or release chromium-containing sludges and wastewater into the untreated environment in order to reduce costs, due to the outdated treatment technologies used in the production of chromates [8]. Cr exists in two main oxidation states: Cr(VI) and Cr(III) [9,10]. Among them, Cr(III) is a crucial trace element for our body, while Cr(VI) is considered one of the most toxic heavy metal ions in surface water because of its high toxicity and mobility [11]. Therefore, the development of technologies to effectively remove Cr(VI) has become a global concern.

Various physical and chemical techniques such as photocatalysis [12], biological treatment [13], electrodialysis [14], membrane filtration [15] and adsorption [16] have been applied for the removal of Cr(VI) from industrial wastewater. Compared with other methods, photocatalytic reduction can be carried out under mild reaction conditions to convert highly-toxic Cr(VI) into low-toxic Cr(III), achieving the essential removal of Cr(VI) [17]. Moreover, solar energy as the energy source for photocatalysis is green, clean and low-cost [18,19]. Therefore, how to improve the photoreduction ability of materials for Cr(VI) has become a hot topic [20]. On the one hand, the visible light utilization and free radical generation of the photocatalysts can be enhanced by molecular design [21,22], structural modulation [23], and heterogeneous structures [24,25]. On the other hand, the mass transfer process can also be enhanced to improve the photocatalytic performance. The extent to which the reaction proceeds can be enhanced by increasing the concentration of reactants or decreasing the concentration of products. According to Equation (1) [26], increasing the concentration of Cr(VI) and the total number of photogenerated electrons on the catalyst surface or decreasing the concentration of Cr(III) can achieve a positive shift in the reduction reaction. However, it is disappointing that there are limited studies on this aspect. Moreover, powder-based photocatalysis have become increasingly inadequate due to the difficulty of recycling [27]. Therefore, there is a need to explore and develop more advanced and sustainable photocatalytic technologies to address these challenges and meet future demands.

Cr_2_O_7_^2−^/2HCrO_4_^−^ + 14H^+^ + 6e^−^ → 2Cr(III) + 7/8H_2_O (1)

Capacitive deionization (CDI), a technique that enables ions in solution to move in a controlled manner, can realize the desired outcome [28]. Under the influence of the electric field, Cr(VI) oxyanions are enriched on the anode surface, while the reduced Cr(III) cations move away from the catalyst anode to vacate the reaction site. Thus, this technique may greatly enhance the photocatalytic mass transfer process as a way to improve the efficiency of Cr(VI) photoreduction.

Herein, we have integrated CDI with photocatalytic technology, denoted as PCS, to achieve electrosorption-assisted photoreduction of Cr(VI). For the photocatalytic material, PAF-54, a nitrogen-rich porous organic polymer (POP) with strong adsorption capacity for anions [29], was chosen. In this work, the adsorption capacity of PAF-54 for Cr(VI) and its adsorption mechanism were investigated. It was then coated on carbon paper (CP) and employed as a photocatalytic electrode for the PCS device. Additionally, a hole scavenger (TA) was introduced to further improve the photocatalytic efficiency and facilitate the removal of Cr(VI). The result was demonstrated that photoreduction efficiency of Cr(VI) was significantly enhanced and excellent stability was maintained under the action of the electric field. For 10 ppm Cr(VI), 100% removal can be achieved in 60 min.

## 2. Results and Discussion

### 2.1. Structural Characterization

**Figure 1:**
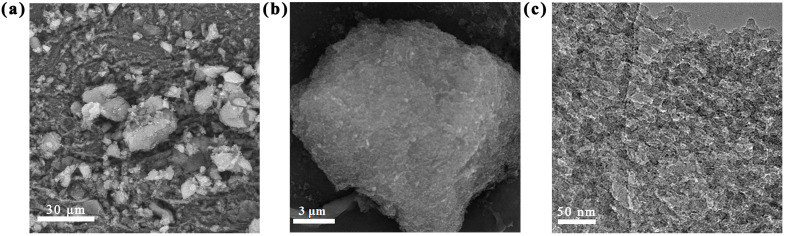
(**a**,**b**) SEM image and (**c**) TEM image of PAF-54.

The successful preparation of PAF-54 was confirmed by FT-IR spectra and solid-state 13C NMR (Figure S1). No distinct diffraction peak in PXRD pattern (Figure S1c), indicating that PAF-54 is an amorphous network, possibly resulting from irreversible coupling reactions [30]. The irregular morphology of the prepared sample was characterized by SEM and TEM (Figure 1), which supported the amorphous nature of PAF-54, consistent with the PXRD results. PAF-54 is formed by the accumulation of complex sheet-like materials, which results in non-dense particles of varying sizes in the SEM images due to the disordered accumulation. The EDS analysis of PAF-54 is shown in Table S1, the PAF-54 skeleton contains up to 58.95% of elemental N, serving as the potential adsorption sites for Cr(VI).

**Figure 2:**
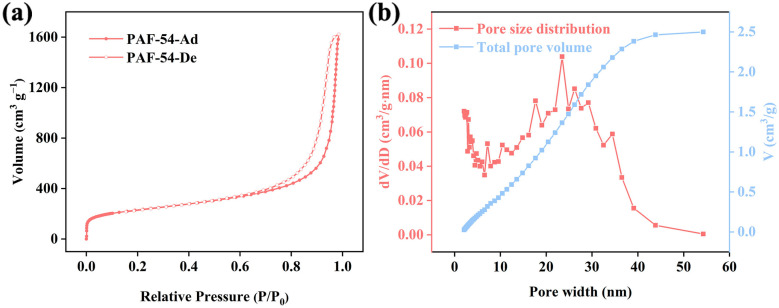
(**a**) Nitrogen adsorption-desorption isotherm, (**b**) pore size distribution.

As shown in Figure 2a, the sorption isotherm exhibits type IV characteristics with an obvious hysteresis loop. In addition, based on the pore size distribution plot (Figure 2b), it is evident that there PAF-54 contains numerous mesopores. Its high specific surface area (795.3 m^2^/g), large pore volume (2.511 cm^3^/g) and suitable average pore size (14.32 nm) are very favorable for adsorption of Cr(VI) oxyanions.

### 2.2. Adsorption Behavior and Mechanism

#### 2.2.1. Influence of pH toward Structure and Adsorption

**Figure 3:**
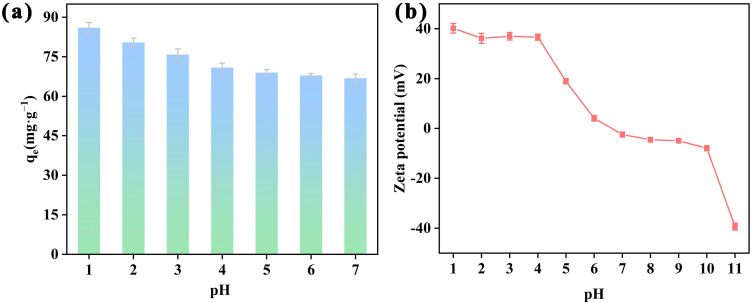
(**a**) Effect of pH on Cr(VI) adsorption of PAF-54. (m/V = 0.25 g/L, *C*_0_ = 75 mg/L, *t* = 24 h) (**b**) The zeta potential of PAF-54 at different pH.

To study PAF-54’s performance for Cr(VI) adsorption, the adsorption behavior of PAF-54 under different pH conditions (1–7) was firstly investigated. The adsorption capacity of PAF-54 for Cr(VI) gradually increased as the pH decreased, reaching a maximum value of 87.16 mg/g at pH = 1 (Figure 3a). This can be attributed to the fact that Cr(VI) exists in various forms at different pH (Figure S2), including HCrO_4_^−^, Cr_2_O_7_^2−^ and CrO_4_^2−^. HCrO_4_^−^ are predominantly present at pH< 6.5, while CrO_4_^2−^ dominates at pH > 6.5 [31]. In addition, the pH of the solution not only changes the forms of heavy metal ions present in aqueous solution, but it also affects the protonation degree and surface charge of PAF-54 [32]. As shown in Figure 3b, at low pH (<7), protonation imparts a significant positive charge on the surface of PAF-54, thus enhancing the interaction with the anionic Cr(VI) and leading to improved adsorption capacity. And with a decrease in pH, the protonation capacity of PAF-54 was enhanced, boosting its adsorption capacity for Cr(VI) oxyanions. In order to simulate the strong acidic condition in electroplating wastewater, in the next series of adsorption experiments, the pH of the Cr(VI) solution was adjusted to 1.

#### 2.2.2. Adsorption Kinetics Study

**Figure 4:**
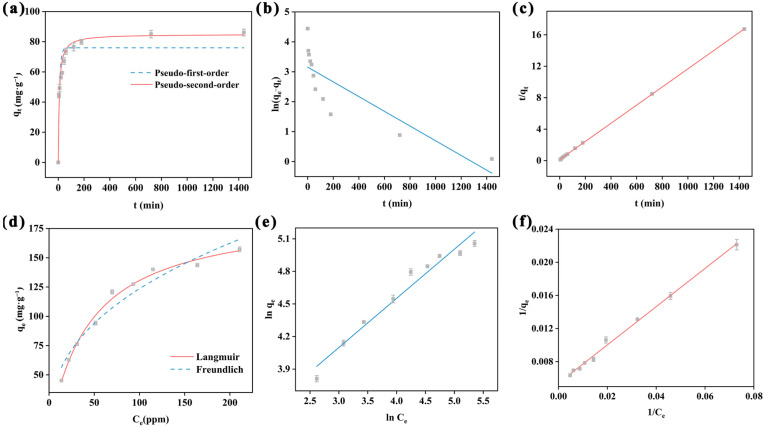
(**a**) Effect of adsorption time on adsorption capacity and the fitted plots obtained by (**b**) pseudo-first-order and (**c**) pseudo-second-order kinetic models. (m/V = 0.25 g/L, *C*_0_ = 75 mg/L, pH = 1) (**d**) Effect of equilibrium concentration of Cr(VI) solution on adsorption capacity and corresponding fitted plots obtained by (**e**) Langmuir, (**f**) Freundlich, (m/V = 0.25 g/L, *C*_0_ = 25-300 mg/L, pH = 1, *t* = 24 h).

Adsorption time is one of the key factors in evaluating the removal rate of adsorbent. Figure 4a shows the adsorption trend of PAF-54 over time. During the early stage of adsorption (*t* < 60 min), the high concentration of Cr(VI) in the solution and the abundance of unoccupied adsorption sites facilitate rapid adsorption of Cr(VI). However, the adsorption rate diminish in the later stage of the adsorption process until the adsorption equilibrium is reached due to the decline in adsorption sites and the concentration of Cr(VI). To obtain a deeper understanding of the adsorption process, the adsorption kinetics of Cr(VI) in PAF-54 were analyzed by classical pseudo-first-order (Equation (2)) and pseudo-second-order kinetic models (Equation (3)).

(2) $$q_{t}=q_{e}\left(1\;-\;e^{-k_{1}t}\right)$$

(3) $$q_{t}=\frac{{q_{e}}^{2}k_{2}t}{1+k_{2}q_{e}t}$$

where *t* is the adsorption time (min), *q_e_* (mg/g) and *q_t_* (mg/g) are the Cr(VI) adsorbed at adsorption equilibrium and the adsorption capacity at *t*, respectively. *k*_1_ (1/min) and *k*_2_ (g/mg·min) are the constants. The pseudo-second-order model and pseudo-first-order of PAF-54 for Cr(VI) adsorption are shown in Figure 4b,c, and the corresponding parameters are shown in Table S2. It is obvious that the former model is more suitable for the adsorption kinetics of Cr(VI) on PAF-54, as indicated by a comparison of the R^2^ values (R^2^ = 0.88129 of pseudo-first-order model, while R^2^ = 0.95768 of pseudo-second-order model) (Table S2). The adsorption process may involve a synergistic effect of electrostatic adsorption and hydrogen bonding between Cr(VI) and adsorbed sites that are uniformly distributed on PAF-54 [29,33,34,35].

#### 2.2.3. Adsorption Isotherm Study

To study the maximum adsorption of Cr(VI) by PAF-54, adsorption isotherm experiments were carried out at 25 °C for different initial concentrations (25-300 mg/L) of Cr(VI). The adsorption capacity of PAF-54 gradually increased with the increase in the initial Cr(VI) concentration of the solution and finally reached the saturated adsorption level with the maximum adsorption capacity (Figure 4d). This is because the contact opportunity of PAF-54 with Cr(VI) increases with the rise in Cr(VI) concentration, leading to higher adsorption. Additionally, higher concentrations of Cr(VI) enhance the driving force of mass transfer, favoring the adsorption of Cr(VI) at equilibrium. Furthermore, Langmuir (Equation (4)) and Freundlich (Equation (5)) isotherm modeling were used to study the adsorption isotherms of Cr(VI) on PAF-54.

(4) $$q_{e}=\frac{q_{m}K_{L}C_{e}}{1+K_{L}C_{e}}$$

(5) $$q_{e}=K_{F}{C_{e}}^{\frac{1}{n}}$$

where *q_m_* (mg/g) denotes the maximum adsorption capacity, *q_e_* denotes the equilibrium adsorption capacity (mg/g), *C_e_* denotes the equilibrium concentration (mg/L), n is an empirical constant, *K_L_* (L/mg), and *K_F_* [(L/mg)^1/n^·mg/g], denote adsorption constants. After calculation (Table S3), the R^2^ of the Langmuir model (R^2^ = 0.99023) is greater than that of the Freundlich model (R^2^ = 0.94536), indicating that the Langmuir model can better describe the adsorption process, which is monolayer adsorption. And the theoretical maximum adsorption capacity can be up to 189.39 mg/g, showing good adsorption capacity of Cr(VI) (Table S4 [36,37,38,39,40,41,42,43,44]).

#### 2.2.4. Adsorption Mechanism

**Figure 5:**
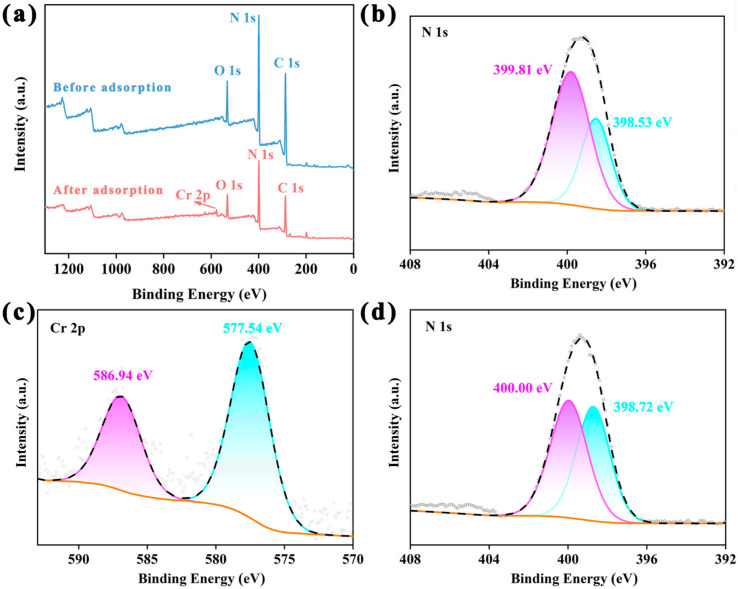
XPS spectra of the sample. (**a**) Survey spectrum of PAF-54 before adsorption and after adsorption; (**c**) Cr 2p of PAF-54 after adsorption; N 1s of PAF-54 (**b**) before adsorption and (**d**) after adsorption.

The adsorption mechanism of PAF-54 was investigated. The presence of Cr peaks on the XPS spectrum and the ICP result (Cr: 8.2%) proved the presence of Cr on PAF-54 after adsorption. And compared with pure Cr_2_O_7_^2−^ [579.88 eV (Cr 2p_3/2_) and 589.18 eV (Cr 2p_1/2_)] [35], a significant decrease in the binding energy occurred (Figure 5c). Meanwhile, comparing the N 1s spectra before and after adsorption, the binding energy increased from 399.81 eV (-NH-) and 398.53 eV (-C=N-) to 400.00 eV and 398.72 eV, respectively, which can be attributed to the surface complexation of Cr (VI) with C = N and N-H on PAF-54 (Figure 5b,d). In addition, comparison of the FT-IR spectra of PAF-54 before and after adsorption (Figure S3) revealed a significant blue shift in the broad peak of N-H from 3418 cm^−1^ to 3430 cm^−1^ after the adsorption of Cr(VI). This shift may be attributed to the formation of hydrogen bonds. In addition, the characteristic band of Cr(VI) is observed at 939 cm^−1^, suggesting that Cr(VI) has been adsorbed onto PAF-54. In addition, based on the zeta potential of PAF-54 and the form of Cr(VI) present in aqueous solution, under low pH conditions, the secondary amines in PAF-54 are easily protonated to form positively charged groups on the adsorbent surface. Meanwhile, Cr(VI) mainly exists in a negatively charged state under acidic conditions. Therefore, the strong electrostatic interaction between the positively charged PAF-54 surface and the Cr(VI) oxyanions leads to a significant adsorption of Cr(VI).

### 2.3. Combination of Photocatalysis and CDI

#### 2.3.1. Characterization of PAF-54@CP for Cathode

While adsorption is a fast and effective method for removing Cr(VI), simply transferring toxic Cr(VI) from one phase to another does not achieve a fundamental removal. Conversion of Cr (VI) to Cr(III) by photogenerated electron reduction provides an useful method to eliminate toxic Cr(VI). In order to fit the actual treatment scene, PAF-54 was coated on carbon paper to be prepared as a device and combined with electric field and hole scavengers to enhance the photocatalytic effect. As shown in the SEM images (Figure S4), the blocky PAF-54 is intercalated in the carbon fibers and the combination with CP enhances the overall visible light absorption of the composite (Figure S5).

#### 2.3.2. Effect of Electric Field for PCS System

**Figure 6:**
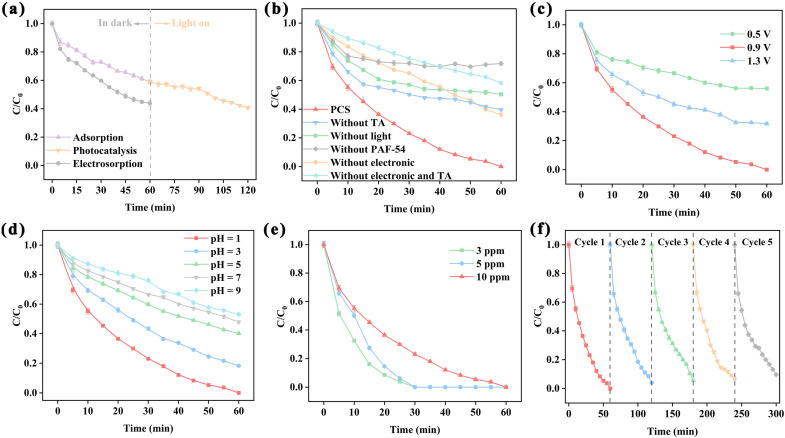
(**a**) Adsorption, electrosorption and photocatalysis of Cr(VI) by PAF-54@CP. ([Cr(VI)]_0_ = 10 ppm, V = 50 mL, pH = 1, E = 0.9 V, [TA] = 0.4 mmol/L) (**b**) The effect of TA, light, electronic and PAF-54 on Cr(VI) removal by PCS system. ([Cr(VI)]_0_ = 10 ppm, V = 50 mL, pH = 1, E = 0.9 V, [TA] = 0.4 mmol/L)The effect of (**c**) voltage ([Cr(VI)]_0_ = 10 ppm, V = 50 mL, pH = 1, [TA] = 0.4 mmol/L), (**d**) pH ([Cr(VI)]_0_ = 10 ppm, V = 50 mL, E = 0.9 V, [TA] = 0.4 mmol/L) and (**e**) Cr(VI) concentration (V = 50 mL, pH = 1, E = 0.9 V, [TA] = 0.4 mmol/L) on Cr(VI) removal. (**f**) Reusability test of PCS system. ([Cr(VI)]_0_ = 10 ppm, V = 50 mL, pH = 1, E = 0.9 V, [TA] = 0.4 mmol/L).

Aided by an electric field, the Cr(VI) oxyanion tends to move towards the anode, thus enhancing the adsorption capacity of PAF-54 for Cr(VI). As shown in Figure 6a, when an additional electric field of 0.9 V was applied, the concentration of Cr(VI) in the solution was reduced to 43.7% of the initial level, which was much higher than that of the static adsorption. This resulted in rapid enrichment of Cr(VI). However, after combining CDI and photocatalysis, its Cr(VI) removal effect was still unsatisfactory, probably due to the weaker ability of PAF-54 to generate reactive radicals (e^−^, •O_2_^−^, etc.). To enhance the photocatalytic effect of PAF-54, TA was added to the PCS system to promote the generation of photogenerated electrons. As expected, the device achieved 100% removal of Cr(VI) within 60 min after the addition of TA (Figure 6b).

It is known that voltage is a crucial parameter for this system [45]. As the voltage increases, the directional movement of ions becomes more intense, leading to improved electrosorption and enhancing the mass transfer process in the photocatalytic process (Figure 6c). However, when the voltage was raised to 1.3 V, the removal effect of Cr(VI) was weakened instead. This may have been the voltage being too high, which led to the electrolysis of water and subsequently affecting the reduction in Cr(VI). Therefore, an optimum potential of 0.9 V was chosen for subsequent experiments. In addition, the absence of any redox peaks in the CV curves suggested that no electrocatalytic process occurred in the process (Figure S6). The electric field only served to enhance adsorption as an adjunct to photocatalysis.

#### 2.3.3. Effect of pH for PCS System

The impact of pH on the removal of Cr(VI) by this PCS system was examined. As shown in Figure 6d, the photocatalytic ability decreases as the pH rises. At pH = 1, Cr(VI) can be completely reduced within 60 min, but when pH = 9, the removal of Cr(VI) is only 47.8%. On the one hand, Cr(VI) mainly exists in the form of Cr_2_O_7_^2−^ and HCrO_4_^−^ under acidic conditions, as indicated in Equations (6) and (7), and a higher H^+^ concentration is favorable for the reduction process. In contrast, under alkaline conditions, mainly Cr(VI) was reduced to Cr(OH)_3_ precipitate, which may deposit on the catalyst surface, thus reducing the photocatalytic activity. In addition, higher OH^−^ concentration is rather unfavorable for the reaction to proceed in the positive direction (Equation (8)) [46]. On the other hand, as investigated above, pH affects the electrostatic adsorption of Cr(VI) by PAF-54. At low pH, PAF-54 exhibits strong adsorption capacity for Cr(VI) oxyanions. The enriched Cr(VI) on the catalyst surface is beneficial to the reduction reaction process towards the positive direction.

Cr_2_O_7_^2−^ + 16H^+^ + 6e^−^ → 2Cr^3+^ + 7H_2_O (6)

HCrO_4_^−^ + 7H^+^ + 3e^−^ → Cr^3+^ + 4H_2_O (7)

CrO_4_^2−^ + 4H_2_O + 3e^−^ → Cr(OH)_3_↓ + 5OH^−^ (8)

#### 2.3.4. Catalytic Activity and Stability for PCS System

Figure 6b shows the effect of each experimental condition on the photocatalytic reduction in Cr(VI) by the PCS system. When any of the factors of TA, light, catalyst or electricity are absent, the catalytic effect is greatly reduced. Due to the synergistic effect of CDI and photocatalysis with TA attached, this PCS process exhibits a robust Cr(VI) photoreduction capability, which is superior to most of the current POPs-based photocatalysts (Table S5 [21,46,47,48,49,50]). For 10 ppm Cr(VI), 100% removal can be achieved in 60 min. Faster degradation can be achieved for lower concentrations of Cr(VI) solutions (Figure 6e). Furthermore, the catalyst showed excellent stability, with no significant decrease in Cr(VI) removal efficiency after five cycles (Figure 6f) and there was not any significant change in PAF-54 on CP from FTIR (Figure S7). 

#### 2.3.5. Possible Mechanism of PCS

**Figure 7:**
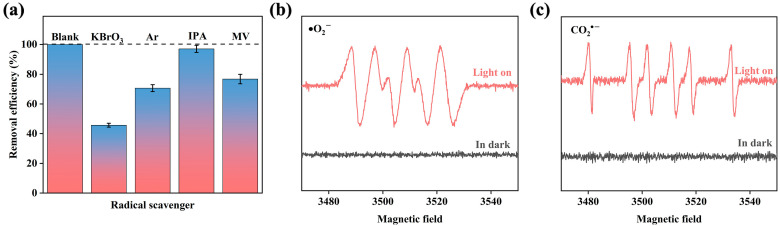
(**a**) Inhibition of different scavengers on Cr(VI)removal. ([Cr(VI)]_0_ = 10 ppm, V = 50 mL, pH = 1, E = 0.9 V, [TA] = 0.4 mmol/L, [KBrO_3_]_0_, [IPA]_0_ and [MV]_0_ = 10 mmol/L) ESR spectra of (**b**) DMPO-•O_2_^−^ and (**c**) DMPO- CO_2_^•−^.

Photogenerated electrons can directly reduce Cr(VI), and other reactive radicals may also participate in the reaction. In order to explore the role of these active substances, scavenging experiments were performed using KBrO_3_, IPA and MV as e^−^, •OH and CO_2_^•−^ scavengers, respectively (Figure 7a). And, argon was introduced into the solution during catalysis to remove oxygen to identify the effect of •O_2_^−^. As shown in the Figure 7a, the inhibition of Cr(VI) reduction was most obvious with the addition of KBrO_3_, and the removal effect could only reach 45.7%, indicating that e^−^ plays a dominant role in this system. On the one hand, e^−^ can directly reduce Cr(VI), and on the other hand, e^−^ may generate •O_2_^−^ with oxygen in the solution, and then further reduce Cr(VI). After passing argon to exclude oxygen, the Cr(VI) removal efficiency of the PCS system was significantly reduced, indicating that the •O_2_^−^ did play a role in the photoreduction process, and the presence of •O_2_^−^ was captured by ESR (Figure 7b). Similarly, free radical sacrifice experiments as well as ESR (Figure 7c) indicated that CO_2_^•−^ radicals also played a larger role in the reaction process. It should be noted that CO_2_^•−^ can be generated by TA after excitation by visible light [51]. This is probably because •OH is a common oxidizing agent and its effect on Cr(VI) reduction is almost negligible.

**Figure 8:**
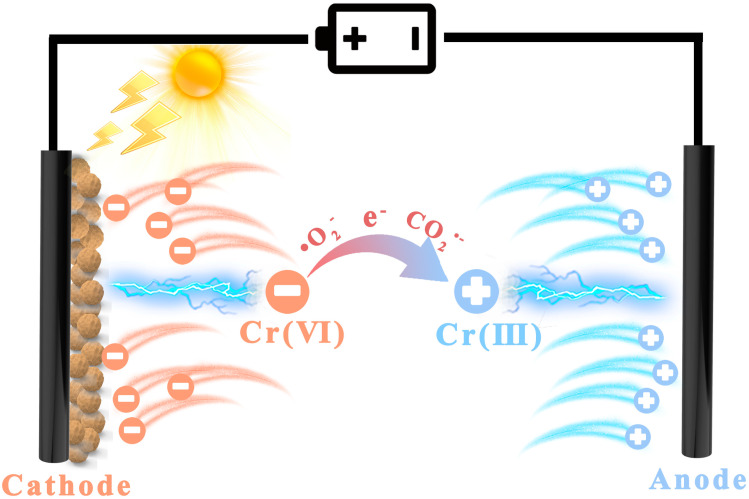
Mechanism of PCS system photoreduction of Cr(VI).

The working mechanism of Cr(VI) removal by PCS system is shown in Figure 8. Firstly, the nitrogen-enriched PAF-54 on the anode can effectively adsorb the Cr(VI) oxyanions through protonation. This enrichment can be further enhanced due to the electric field effect. Later, when PAF-54@CP is exposed to visible light, electrons and •O_2_^−^ are produced on its surface and the Cr(VI) oxyanions enriched on the catalyst surface are reduced to Cr(III) cations and driven away from the catalyst surface by the electric field, leaving more active sites on the catalyst. Notably, the addition of TA promotes the generation of photoelectrons, and the generation of CO_2_^•−^ for direct reduction in Cr(VI). By increasing the concentration of Cr(VI) on the catalyst surface and the number of reactive radicals, supplemented by the decreased concentration of Cr(III), the reaction equation is promoted to proceed positively, accelerating the photoreduction of the highly-toxic Cr(VI) to the low-toxic Cr(III).

## 3. Materials and Methods

### 3.1. Synthesis of PAF-54 

According to the literature (Figure S2) [52], 553.2 mg of cyanuric chloride was dissolved in 30 mL of DMSO solution in a 100 mL beaker to obtain a clarified solution 1. 378.3 mg of melamine was dissolved in 30 mL of DMSO solution, and the 0.4 mL of triethylamine was added to obtain a clarified solution 2 with a molar ratio of melamine and cyanuric chloride of 1:1. Under nitrogen atmosphere, the solution 2 was transferred to a three-necked flask. Then, solution 1 was slowly added to solution 2 during stirring at 300 rpm at room temperature, and a white precipitate gradually appeared. And the reaction was kept at 150 °C for 24 h to obtain a milky white suspension. After cooling to room temperature, the resulting egg-white precipitate was filtered and washed thoroughly with excess DMSO, H_2_O and MeOH, and dried under vacuum at 25 °C.

### 3.2. Preparation of PCS Anode Electrode

Firstly, the commercially available carbon paper was hydrophilically treated. As follows, it was cut to the size of 2.5 cm × 3 cm, and strong acid (HNO_3_) and strong base (KOH) were added sequentially, and the reaction was carried out at 95 °C for 4 h each. Afterwards, it was washed with H_2_O until the CP surface was neutral. Added 40 mg of PAF-54 and 10 mg of PVDF to the mortar and ground for 30 min until PAF-54 and PVDF are well mixed, then added 1 mL NMP (N-Methyl pyrrolidone) and stirred overnight to obtain a uniform PAF-54 slurry. Finally, coated it onto carbon paper and dried at 65 °C.

### 3.3. Cr (VI) Adsorption Experiment 

The solutions were prepared by diluting K_2_Cr_2_O_7_ in H_2_O. And the adsorbed solution was filtrated through a 0.22 μm filtrating diaphragm. Among them, the initial pH of 75 mg/L of Cr(VI) solution was adjusted to 1.0–7.0 to detect the effect of pH value. The adsorption kinetics were studied by adding 5 mg of sample to 20 mL of Cr(VI) solution (75 mg/L), and the concentration of residual Cr(VI) in the solution with different adsorption times was monitored by using the diphenylcarbazide (DPC) method. For the adsorption isotherm, 5.0 mg of sample was added to 25–300 mg/L of Cr(VI) solution. After reaching equilibrium, the concentration of residual Cr (VI) in the solution was measured. Three parallel experiments were conducted for each data. The equilibrium adsorption capacity (*q_e_*, mg/g) is calculated using Equation (9).

(9) $$q_{e}=\frac{\left(C_{0}-C_{e}\right)V}{m}$$

where *C*_0_ denotes the initial concentration of Cr(VI) (mg/L), *V* is the volume of Cr(VI) solution (L), and *C_e_* is the equilibrium concentration of Cr(VI) (mg/L), and *m* is the mass of PAF-54 (g).

### 3.4. PCS System for Cr (VI) Removal

The experiments of Cr (VI) removal were carried by using a standard three-electrode system. In this case, PAF-54@CP, Ag/AgCl electrode and graphite carbon rod were used as a photocatalytic electrode, reference electrode and counter electrode, respectively. Generally, the target solution was 50 mL of 10 ppm Cr(VI) solution and the pH = 1. Then it was irradiated with a 300 W xenon lamp (PLS-SXE300+, Perfect Light, Beijing, China) and a voltage of 0.9 V was applied. After the reaction period, 200 μL of the solution was removed from the PCS system for analysis.

## 4. Conclusions

In summary, the combination of CDI and photocatalysis was utilized to achieve efficient photoreduction of Cr(VI). PAF-54 as an anode catalyst, by virtue of its structure containing a large number of nitrogen elements with inherent Brønsted basic functionality, effectively enriched protons and Cr(VI) oxyanions on its surface under acidic conditions. This enrichment process was further enhanced by the application of an electric field, leading to an increase in the detachment of Cr(III) cations. As a result, the Cr(VI) photoreduction capacity was greatly improved. Furthermore, the addition of TA promoted the generation of photogenerated electrons and CO_2_^•−^, which further strengthened the removal efficiency of the system for Cr(VI). This study provides an effective method for the removal of Cr(VI) by metal-free-based photocatalysts.

## Data Availability

Data will be made available on reasonable request. Data is contained within the article and supplementary material.

## Supplementary Materials

The following supporting information can be downloaded at: https://www.mdpi.com/article/10.3390/ijms25052832/s1.
