# Supplementary material for: Enhancement of Mass Transfer Process for Photocatalytic Reduction in Cr(VI) by Electric Field Assistance
Source: Int J Mol Sci. 2024 Feb 29;25(5):2832. doi: 10.3390/ijms25052832 (PMC10931939; doi:10.3390/ijms25052832)
Supplement: Supplementary file 1 [file ijms-25-02832-s001.zip › ijms-2881179-supplementary.pdf]

Supporting Information

Enhancement of Mass Transfer Process for Photocatalytic Reduction in Cr(VI) by Electric Field Assistance

Xi Feng [1,†], Yonghui Lin [2,†], Letian Gan [2], Kaiyuan Zhao [2], Xiaojun Zhao [2,*], Qinhe Pan [2] and Guohua Fu [3,*]

1 School of Ecology and Environment, Hainan University, Haikou 570228, China
2 School of Chemistry and Chemical Engineering, Hainan University, Haikou 570228, China
3 Management School, Hainan University, Haikou 570228, China

Materials

All the materials and chemical reagents were commercially available. Cyanuric chloride, melamine, triethylamine (Et_3N), dimethyl sulfoxide (DMSO), tartaric acid (TA), polyvinylidene difluoride (PVDF), N-Methyl pyrrolidone (NMP) and Potassium dichromate ($K_2Cr_2O_7$) were purchased from Shanghai Aladdin Biochemical Technology Co., Ltd.

Characterization

Fourier transform infrared (FT-IR) spectroscopy was measured by Shimadzu IRAffinity-1 S infrared spectrometer. The crystal structures were examined by Powder X-ray diffraction (Rigaku Miniflex 600 X-ray diffractometer). Thermogravimetric analysis (TGA) was recorded on a Rigaku Therma plus EVO2 TG-DTA 8122 instrument. Solid state ^{13}C CP/MAS NMR was carried out using a Bruker Avance III HD in 400MHz. Scanning electron microscope (SEM) images and transmission electron microscope (TEM) images were taken on SEM, Phenom Scientific ProX G5 and JEM-2100. Elemental distribution measured by Energy Dispersive Spectrometer (EDS). N_2-adsorption isotherms were obtained at 77 K using an JW-BK112. XPS was recorded on a Thermo ESCALAB 250Xi with Al Kα (1486.6 eV). A Particle Analyzer (SurPASS 3) was used to measure the Zeta potential. The absorbance of Cr(VI) solution was tested with a UV-2700 UV-Vis spectrophotometer and the Cr(VI) concentration was calculated. All electrochemical measurements were conducted on the CHI 660E electrochemical workstation.

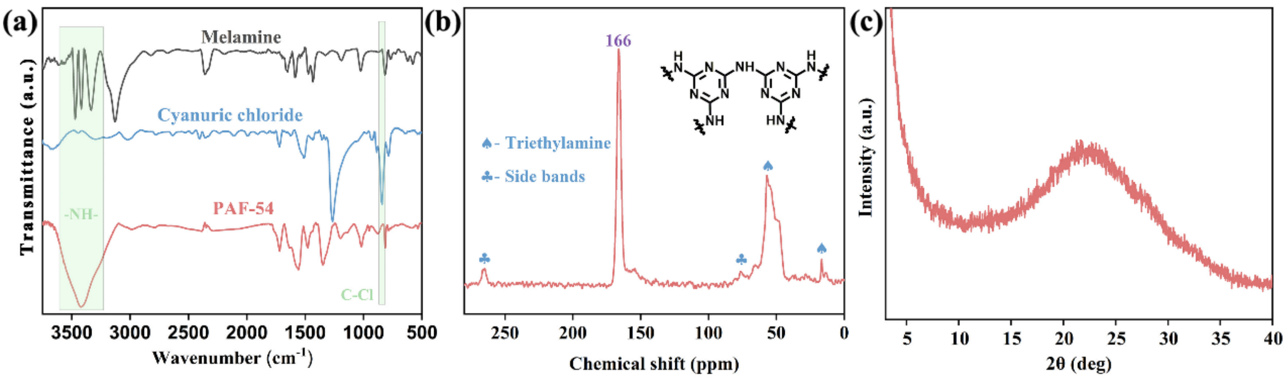

Figure S1. (a) FTIR spectra of cyanuric chloride, melamine and PAF-54. (b) ^{13}C CPMAS NMR spectra and (c) PXRD pattern of PAF-54. In the IR spectra of PAF-54, the peaks at around 1561 cm^{-1}, 1473 cm^{-1} and 1342 cm^{-1} indicate the presence of triazine rings. And the breathing mode vibration of the triazine ring was also shown as a sharp peak at 813 cm^{-1}. The clear observation of a broad band above 3388 cm^{-1}, belonging to the secondary amine (instead of multiple bands similar to those of the amino group in melamine), as well as the disappearance of the stretching vibration of the C-Cl bond at 841 cm^{-1}, represent the complete reaction of melamine and cyanuric chloride. Besides, the strong signal at 166 ppm in the ^{13}C CP-MAS NMR spectrum is from the carbon of the triazine ring, further confirming the successful preparation of PAF-54. Among them, the peak observed at 56 ppm may be attributed to the catalyst (triethylamine).

Table S1. Characteristics of PAF-54.

**Pore parameter**			**EDS mapping (%)**		
S_{BET} (m^2/g)	**Average pore size (nm)**	V_{tot} (cm^3/g)	**C**	**N**	**O and other elements**
795.5	14.32	2.511	37.93	58.95	3.12

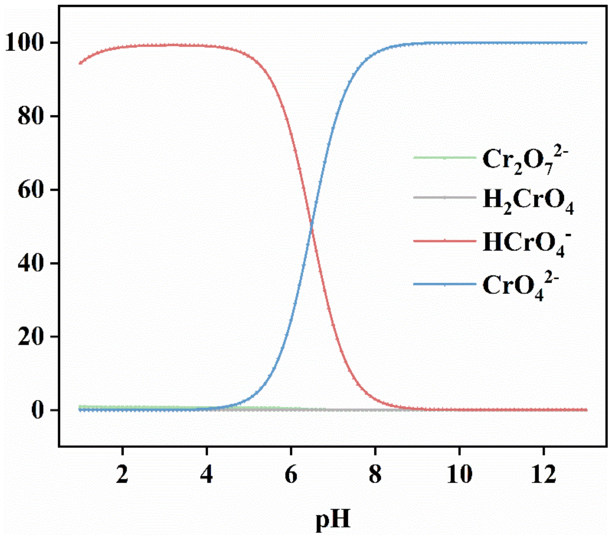

Figure S2. The percentage of different Cr (VI) species in aqueous solutions (C_0 = 10 mg·L^{-1})

Table S2. Adsorption kinetics parameters of the PAF-54 obtained through the Pseudo-first-order and Pseudo-second-order models.

Experimental data (mg/g)	Pseudo-first-order model			Pseudo-second-order model		
	q_e	k_1	R^2	q_e	k_2	R^2
87.16	75.97±3.60	0.09998±0.02378	0.88129	84.95±2.48	0.00148±0.00010	0.95768

Table S3. Isotherms adsorption parameters associated with the Langmuir, Freundlich, isotherms for adsorption of Cr (VI) by the PAF-54.

Langmuir model			Freundlich model		
q_m	K_L	R^2	n	K_F	R^2
189.39±5.67	0.0221±0.0019	0.99022	0.39470±0.03862	20.067±3.60	0.94536

Table S4. Summary of various porous adsorbents for Cr (VI).

Entry	Material	pH	solid-liquid ratio (g/L)	concentration range	Q_m (mg/g)	Ref.
**1**	iPOP-Cl	7	1	147-515 ppm	178	[36]
**2**	BUT-39	3	1	25-500 ppm	107	[37]
**3**	SCNU-Z1-Cl	N/A	0.2	5-100 ppm	142	[38]
**4**	CCD-PCP	3.5	0.05	0-500 ppm	216	[39]
**5**	MCC-PVIM	3	1	21-211 ppm	139	[40]
**6**	PVIm-6-SCD	2	0.4	52-519 ppm	279	[41]
**7**	CPN-tpm-Cl	2	0.2	5-250 ppm	183	[42]
**8**	aMOC-1	3	0.2	5-200 ppm	101	[43]
**9**	aMOC-2	3	0.2	5-200 ppm	203	[43]
**10**	CON-1	N/A	0.83	37-370 ppm	146	[44]
**11**	**PAF-54**	**1**	**0.25**	**25-300 ppm**	**189**	**This work**

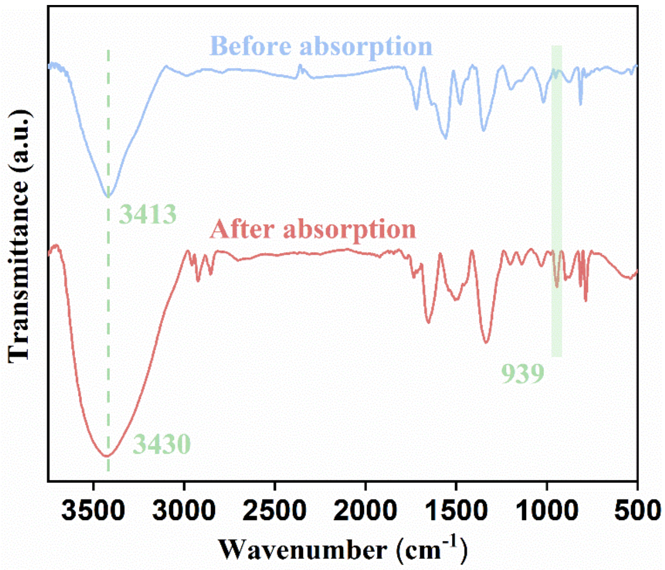

Figure S3. FTIR spectra of PAF-54 before and after adsorption.

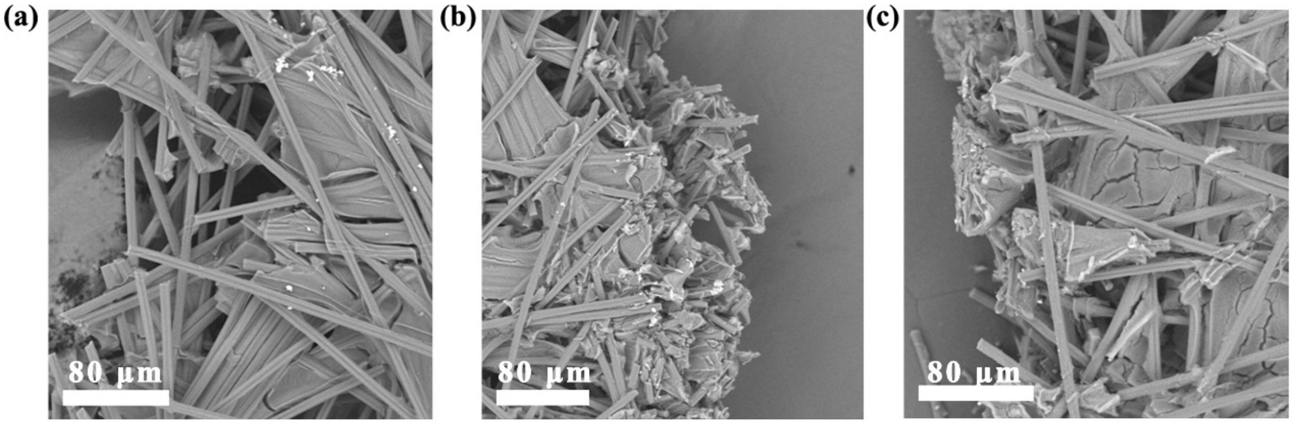

Figure S4.SEM images of (a) CP, (b) PAF-54@CP and (c) PAF-54@CP after photocatalysis.

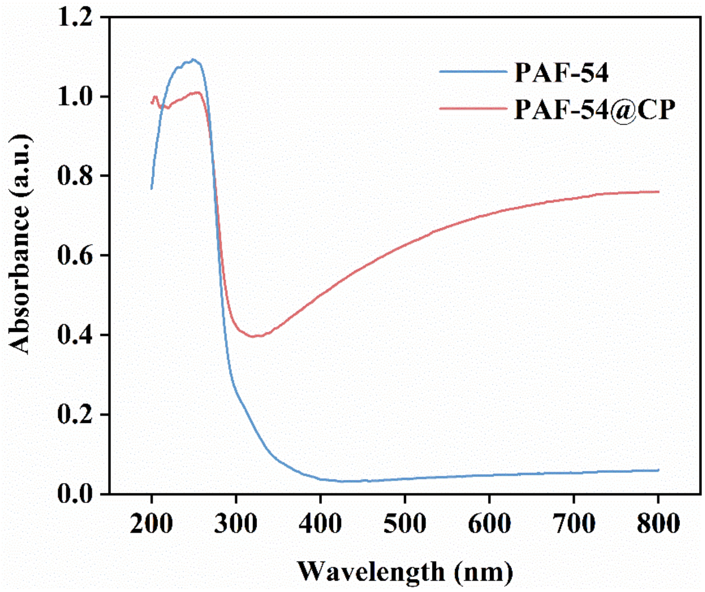

Figure S5. UV-vis diffuse reflection spectra of PAF-54 and PAF-54@CP

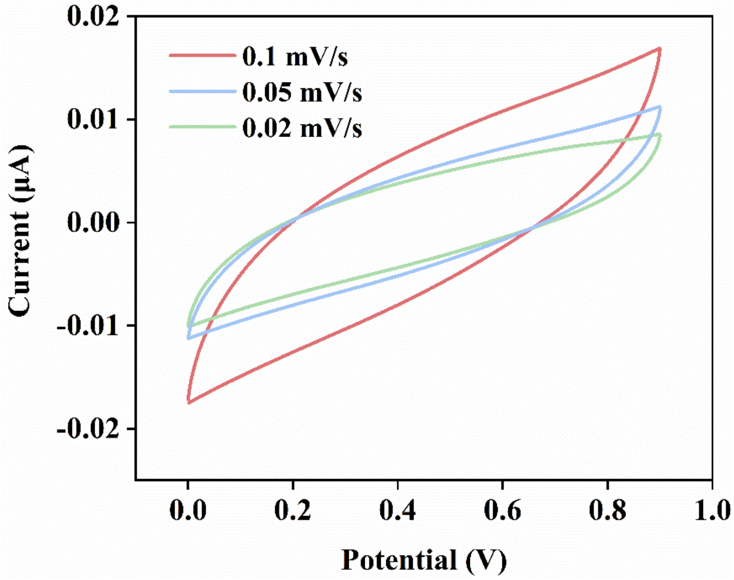

Figure S6 CV curves of PCS device at various scan rates.

Table S5. Photocatalytic performance of various POPs towards Cr (VI) removal.

Entry	Material	pH	$[Cr(VI)]_0$	Hole scavenger	Time (min)	Removal ratio (%)	Ref.
**1**	COF-H	3	5 ppm, 50 mL	EDTA	60	98	[47]
**2**	COF-Br	3	5 ppm, 50 mL	EDTA	60	95	[47]
**3**	COF-OMe	3	5 ppm, 50 mL	EDTA	60	100	[47]
**4**	BiOBr/TzDa COF	2.1	5 ppm, 50 mL	Methanol	60	98	[46]
**5**	TPB-BT-COF	6.1	5 ppm, 10 mL	Phenol	60	99	[48]
**6**	Au@C2	N/A	9 ppm, 30 mL	IPA	120	98	[21]
**7**	TzDa/Ag/AgBr	3.5	5 ppm, 50 mL	TC	90	98	[49]
**8**	JOU-6	3	9 ppm, 30 mL	IPA	100	87	[50]
**9**	JOU-7	3	9 ppm, 30 mL	IPA	100	95	[50]
**10**	**PAF-54**	**1**	**5 ppm, 50 mL**	**TA**	**30**	**100**	**This work**
**11**	**PAF-54**	**1**	10 ppm, 50 mL	**TA**	**60**	**100**	**This work**

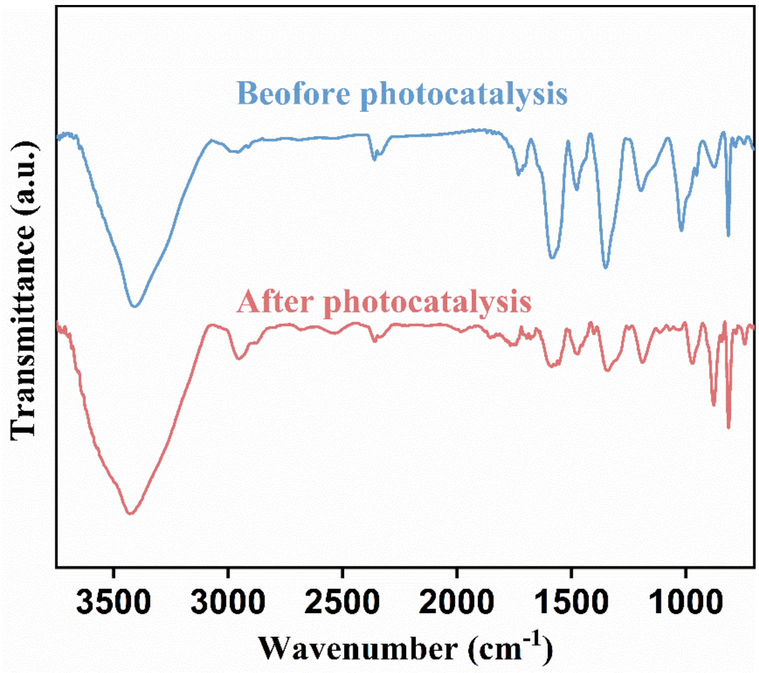

Figure S7. FTIR spectra of PAF-54 before and after photocatalysis.
